# Biochemical Characterization of Early Osteoarthritis in the Ankle

**DOI:** 10.1155/2014/434802

**Published:** 2014-02-13

**Authors:** Hagen Schmal, Gian M. Salzmann, Elia R. Langenmair, Ralf Henkelmann, Norbert P. Südkamp, Philipp Niemeyer

**Affiliations:** Department of Orthopaedic and Trauma Surgery, University of Freiburg Medical Center, Hugstetter Street. 55, 79106 Freiburg, Germany

## Abstract

*Purpose.* Reliable data about *in vivo* regulation of cytokines in early ankle osteoarthritis (OA) are still missing. *Methods.* 49 patients with a mean age of 33 ± 14 years undergoing an arthroscopy of the ankle with different stages of chronic OA were prospectively included in a clinical trial. Lavage fluids were analyzed by ELISA. Additionally, clinical parameters and scores (FFI, CFSS, and AOFAS) were evaluated and supplemented by the Kellgren Lawrence Score (KLS) and the ankle osteoarthritis scoring system (AOSS). *Results.* ICRS grading of cartilage damage, previous operations, and duration of complains were strong indicators for OA progress and showed correlations to age, clinical scores, validated KLS, and AOSS (*P* < 0.04). Systemic and intraarticular inflammatory parameters were low in all patients. Biochemically, aggrecan and BMP-7 positively indicated OA with statistically significant associations with duration of symptoms, FFI, AOFAS, and KLS (*P* < 0.04). In contrast, BMP-2 levels showed statistically significant negative correlations to aggrecan or BMP-7 concentrations, which is in line with the negative association with ICRS score and KLS and the positive correlation with FFI (*P* < 0.03). *Conclusions.* We were able to identify different key markers of OA in the ankle as aggrecan, BMP-7, and BMP-2, offering starting points for new ways in diagnostics and interventional strategies.

## 1. Introduction

The ankle is one of the most biomechanically challenged joints during stance phases and walking [1]. The combination of such high demands with certain pathologies disturbing the normal joint symmetry as ankle fractures or the normal biochemistry as hemophilia predestines this joint to develop an osteoarthritis (OA). Although in the majority of patients with ankle fractures the restoration of anatomy is possible the risk to develop posttraumatic OA is overall almost 40% [2]. Besides fracture complexity, increasing body mass index, age over 30 years, and length of time since surgery were identified as significant risk factors. This indicates that the understanding of the pathological synovial cytokine regulation within the joint cavity following biochemical or biomechanical impacts may be crucial to develop sufficient interventional strategies accompanying the conventional treatment methods. Since degeneration of cartilage is one of the decisive steps in OA development, dysfunction and disturbed control of mediators influencing cartilage metabolism following different pathologies of the ankle may be hypothesized [3]. Furthermore, particular regulatory patterns may be suspected, because studies have shown that biomechanics and functional reaction of chondrocytes are ankle specific [4, 5]. The course of OA is characterized by certain arthroscopic findings, clinical symptoms, and radiographic changes [6, 7]. This study aimed to connect the measurement of intra-articular cytokine levels with clinical and radiographic quantitation of OA-related joint alterations. In order to cover a representative spectrum of different proteins or mediators, synovial concentrations of BMP-2, BMP-7, endoglin (part of the BMPR-1A complex), bFGF and IGF-1 and its receptor as marker of intrinsic cartilage repair, IL-1*β* and MMP-13 as a marker of inflammation, aggrecan as an integral part of the extracellular matrix (ECM), and the total protein content were determined.

Osteoarthritic progression is thought to be associated with the upregulation of bFGF expression, which may be caused by the fact that cartilage injury leads to release of bFGF from chondrocytes [8]. The effects of bFGF on chondrocyte proliferation and differentiation are controversial, leading to the conclusion that bFGF is necessary for a functional balance during repair processes [9]. bFGF has been shown to inhibit the anabolic effect of IGF-1 [10], a cytokine with immanent importance as a promoter of growth and matrix synthesis by chondrocytes in healthy articular cartilage. IGF-1 enhances aggrecan synthesis by articular cartilage cells or explants, which has been demonstrated in cell culture experiments and using in vivo animal models [11]. IGF-1 is also produced by chondrocytes and stored within the extracellular matrix of cartilage, probably bound to proteoglycans, particularly to the cell-surface located syndecans and the IGF-1 binding proteins [12, 13]. IL-1*β* is one of the best described inflammatory mediators that also disturbs the homeostasis of the extracellular matrix (ECM) of articular cartilage in osteoarthritis. Downstream IL-1 features include induction of collagenases, proteoglycanases, and matrix metalloproteinase (MMP) activities as MMP-13 [14], the expression of inducible nitric oxide synthase (iNOS), and the secretion of prostaglandin E2, IL-6, and IL-8 [15]. IL-1 is also capable of reducing the production of cartilage-specific macromolecules, including aggrecan and type II collagen, through modulation of the transcription factors Sp1 and Sp3 [16]. Aggrecan is a large proteoglycan, represents an integral part of the ECM, and is effectively able to neutralize compressive forces. Aggrecan is situated in the interstices of the collagen meshwork, forming large complexes on the basis of an interaction with hyaluronan and link proteins. Aggrecanase mediated degradation of aggrecan is a paramount in development of osteoarthritis (OA) [17]. BMP-2 and BMP-7 play a significant role in skeletal development and are potent inducers of bone formation [18]. BMP-2 is able to promote chondrogenesis in human mesenchymal stem cells [19]; BMP-2 coating of scaffolds resulted in mature cartilage formation using either mesenchymal stem cells or amplified chondrocytes [20]. Furthermore, mechanical stress was found to upregulate BMP-2 as well as BMP-2 signaling in human cartilage explants [21], indicating a role for BMP-2 in natural cartilage reparative processes. BMP-7 exhibits characteristics as an anabolic factor in cartilage metabolism [22]. BMP-7 was able to enhance synthesis of extracellular matrix components and to promote cartilage repair. BMP-2, BMP-7, and BMPR-1A were expressed in cartilage and synovia of human knees with focal cartilage lesions [23]. BMP-2 seems to play an important role in surgically induced cartilage repair, because synovial expression correlated with the clinical outcome [24]. BMPR-1A was associated with the development of OA, because expression was diminished in repair cartilage of human specimens. This was also shown in patients with osteochondritis dissecans (OCD) [25].

Besides changes of the intra-articular milieu progress of OA may be evaluated by different clinical factors. For example, duration of complains, previous operations, characterization of associated cartilage lesions by size and depth, and grading of OCD have been described as reliable parameters [26]. These data are supplemented by different clinical scores providing a summary of region-specific function [27, 28]. OA related changes are also reflected by different imaging techniques. Therefore, semiquantitative radiographic scores evaluating changes in conventional X-rays (Kellgren Lawrence Score (KLS) [29]) and MRI (ankle osteoarthritis scoring system (AOSS) [30]) were included in the analysis.

Inflammatory reactions and cartilage repair mechanisms may play a role in the progress of OA following cartilage damage. The purpose of our study was to quantify the amount of potentially chondrodestructive and chondroprotective cytokines present in the ankle and to determine if the cytokine profiles correlate with the amount of cartilage destruction noted radiologically, arthroscopically, and by determination of the clinical ankle function.

## 2. Methods

### 2.1. Study Design

The study was approved by the Ethical Board of the University of Freiburg (AN-EK-FRBRG-335/08) and registered at the German Clinical Trials Register (CORRCYT, DRKS00000365).

49 patients were enrolled in a prospective clinical trial between November 2009 and May 2011 as previously described [30]. Patients were included in case of fulfilment of the following criteria: performance of an arthroscopy of the ankle, agreement to participate in the study by patients and/or parents in case of patients <18 years, and age >10 years and <65 years. Exclusion criteria were alcohol or drug abuse, mental retardation with incapability to complete the necessary self-reports, and infection.

### 2.2. Characterization of Patients

The average age was 32.6 ± 13.6 years; the ratio male/female was 31/18 (63%/37%). 15 patients were treated operatively before (31%). 15 patients (31%) were smoker and 34 nonsmoker (69%). The average body mass index (BMI) was 25.4 ± 4.5. 28 patients had an arthroscopy because of osteochondritis dissecans (OCD); further relevant diagnoses were tibiotalar impingement and osteophytes (*n* = 11), ligament instability (*n* = 2), removement of loose joint bodies (*n* = 3), chondromalacia (*n* = 5), and cysts (*n* = 1). Diagnoses of OCD were confirmed by two radiologists and two orthopaedic surgeons; multiple diagnoses were possible.

### 2.3. Specimen Collection

Synovial lavage fluids of ankles of patients undergoing an arthroscopy were intraoperatively collected. Before starting the arthroscopy, 20 mL of sterile ringer solution was instilled into the joint cavity. The fluid was mixed within the joint by repeated passive flexion extension and repeated manipulation of the posterior and anterior ankle regions and then was aspirated. This procedure was described before for knees [31] and ankles [30]. The aspirated volume reproducibly ranged between 8 and 13 mL. Specimens were centrifuged in order to separate the cells and then stored frozen at −80°C until being analyzed [30]. The method was validated by correlation of cytokine concentrations in joint effusions with lavage levels of the same ankle joints confirming statistically significant associations (*P* < 0.0001 for total protein content).

### 2.4. ELISAs for BMP-2, BMP-7, Endoglin, bFGF, IGF-1, IGF-1R, IL-1*β*, MMP-13, Aggrecan, and BCA (Bicinchoninic Acid) Protein Assay

In order to measure concentrations of the indicated proteins, commercially available ELISA kits provided by R&D Systems (Wiesbaden-Nordenstadt, Germany) for BMP-2, BMP-7, endoglin, bFGF, IGF-1, IGF-1R, and IL-1*β* and BioSource (BioSource Deutschland GmbH, Solingen, Germany) for aggrecan were used according to the manufacturers' instructions. Briefly, the assay employs the quantitative sandwich enzyme immunoassay technique. A specific MAb was precoated onto a microplate. Supernatants were applied to the wells and, after washing, an HRP-conjugated specific Ab was added to the wells. Following the next wash, colour development was proportional to protein concentration and was calculated by comparison with a standard. A colorimetric method was used in order to quantify total protein amount in the lavage fluids. The bicinchoninic acid (BCA) assay was available in kit form Pierce (Rockford, Ill, USA) and was used according to the manufacturers' instructions.

### 2.5. Assessment of Radiographic Scores

#### 2.5.1. Ankle Osteoarthritis Scoring System (AOSS)

In order to quantify the OA related changes in the ankles by MRI, the AOSS was used as previously described [30]. The following criteria were assessed.

1. Depth of cartilage damage: grade 0 (0 points): no, grade 1 (1 point): <50% of total cartilage depth, grade 2 (2 points): >50%, grade 3 (3 points): full thickness cartilage defects. The depth of cartilage loss was qualitatively rated in relation to the height of the adjacent intact cartilage or the expected, normal cartilage contour. In doubt, the sagittal T2-weighted sequence was used for the final decision.
2. Defect of the subchondral bone: grade 0 (0 points): no, grade 1 (1 point): minimal (<2 mm), grade 2 (2 points): moderate (2–5 mm), grade 3 (3 points): severe (>5 mm). The depth of the osseous component of the osteochondral defect was scored by estimating the distance between the actual osteochondral defect and the extrapolated subchondral cortex mainly based on evaluation of the coronary or sagittal T1-weighted sequences.
3. Osteophytes: grade 0 (0 points): no, grade 1 (1 point): minimal (<3 mm), grade 2 (2 points): moderate (3–5 mm), grade 3 (3 points): severe (>5 mm). Size was measured from the base to the tip of the osteophyte; baseline was defined as the natural course of the bone.
4. Subchondral cysts (largest diameter): grade 0 (0 points): no, grade 1 (1 point): minimal (<3 mm), grade 2 (2 points): moderate (3–5 mm), grade 3 (3 points): severe (>5 mm). Subchondral cysts were defined as structures of high signal intensity on T2-weighted images in the cancellous bone underlying the joint cartilage.
5. Bone marrow edema (largest diameter): grade 0 (0 points): no, grade 1 (1 point): minimal (<5 mm), grade 2 (2 points): moderate (5–20 mm), grade 3 (3 points): severe (>20 mm). Bone marrow edema was assessed as an area of increased signal intensity on T2-weighted images in the subchondral cancellous bone.
6. Anterolateral or anteromedial meniscoid: 0 points: no, 1 point: yes. MR images were assessed for appearance of pathological anterolateral or anteromedial soft tissue structures.
7. Effusion: 0 points: no, 1 point: yes. If more than a small, physiological sliver of synovial fluid was observed in the T2 images, joint effusion was assumed to be present.
8. Loose joint bodies: 0 points: no, 1 point: yes.
9. Synovitis: 0 points: no, 1 point: yes. Synovitis was evaluated on sagittal images and was reflected by thickening and irregularity of the normally pencil-thin rim of high signal intensity synovium.
10. Soft tissue cysts (Baker cyst analog): 0 points: no, 1 point: yes. These structures may be considered as excrescences originating from the joint capsule. They are depicted as a circumscribed mass with intermediate signal intensity on proton density-weighted and high signal intensity on T2-weighted sequences and are usually observed in the triangle of calcaneus and Achilles tendon.

There are 5 major (1–5) and 5 minor (6–10) criteria. The major criteria are evaluated with up to 3 points and the minor criteria with up to 1 point, respectively. The range of the total score is from 0 to 20 points. The evaluation of scores was done by two different orthopaedic surgeons dedicated to knee- and ankle surgery. Both observers were masked to the patients' biometrical data and were trained using the scoring form. Validation of the score has been previously described [30].

#### 2.5.2. Kellgren Lawrence Score (KLS)

This score has been assessed as described before [29] using an anterioposterior and a lateral view of plain radiographs of the ankle. Mode of evaluation was the same as described for the AOSS. Validation of the score has been previously described [30].

#### 2.5.3. Evaluation of Clinical Scores

The following clinical scores describing the function of foot and ankle were evaluated within 14 days before the operation in order to quantify a possible loss of performance. The foot function index (FFI) was introduced by Budiman-Mak et al. [32] and used in the validated german version published by Naal et al. [33]. The calcaneal fractures scoring system according to Kerr (CFSS) was originally published in order to evaluate the function following calcaneal fractures [27]. Since then, it was used in multiple settings describing function of foot and ankle. Furthermore, the ankle-hindfoot scale (AOFAS), one of the most used scores evaluating the function of foot and ankle with special regard to the lower and upper ankle joint, was used [28]. Both last scores were applied using the translated german and validated version [34]. In order to provide comparability with other studies, three different and region-specific scores were evaluated. ICRS score for grading of cartilage damage was determined as previously described [31] during arthroscopy by the surgeon.

### 2.6. Statistics

All values were expressed as mean ± standard deviation if not otherwise indicated. Correlations were determined by calculating the Spearmen coefficient (*ρ*) for the predominantly not normally distributed values. A cluster analysis was used to reasonably distribute the values in different groups. Based on the different clusters *post hoc* statistics (Kruskal-Wallis *H*-test) were used to analyze statistical significances between the grouped cytokine levels. Individual group means of scores were compared with the rank sum *U*-test. Statistical significance was defined when *P* < 0.05.

## 3. Results

### 3.1. Epidemiology and Characterization of Patients Undergoing Biochemical Analysis

The majority of included patients had no or mild signs of OA, which is reflected by the KLS distribution. 84% [35] of the patients were evaluated with a KLS of 0, 1, or 2, and only 16% [8] were scored higher. This distribution slightly differed from the ICRS score that was ranked between 0 and 2 in 63% [31] and higher in 27% [18], correlating with full thickness defects. The mean duration of symptoms was 40.4 ± 6.2 months, indicating the chronic state of OA. The ICRS score, reflecting the degree of cartilage damage, statistically significantly correlated with age (*P* = 0.03), all clinical scores (FFI: foot function index, CFSS: calcaneal fractures scoring system according to Kerr, AOFAS: ankle-hindfoot scale, *P* < 0.02), KLS, and validated AOSS (*P* < 0.001) (Table 1). The performance of previous operations statistically significantly correlated with a higher ICRS score, a higher defect size, duration of symptoms, age, all clinical scores (FFI, CFSS, and AOFAS), KLS, and AOSS (*P* < 0.03). Although body mass index (BMI) did not show correlations to ICRS grading of cartilage damage or duration of symptoms, there were statistically significant associations with age (<0.001), all clinical scores (FFI, CFSS, and AOFAS, *P* < 0.01), AOSS, and KLS (*P* < 0.04). Overall, the coherence of the clinical data is confirmed by these associations.

### 3.2. Association of Parameters of OA and Biochemical Markers

**Figure 1:**
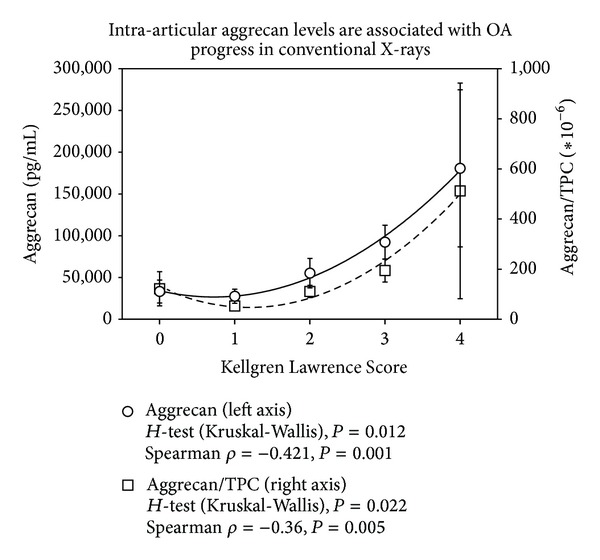
Increasing intra-articular levels of aggrecan are observed with progress of OA measured by radiological changes of the ankle (Kellgren Lawrence Score). Results of the polynomial regression analysis, the Spearman correlation, and the *post hoc* statistics (Kruskal-Wallis *H*-Test) are indicated for absolute aggrecan levels and concentrations in relation to the total protein content (TPC) on the bottom. Polynomial regression with degree of 2 gives an *R* ^2^ = 0.9941 for absolute and *R* ^2^ = 0.9811 for relative concentrations; values are shown with ±SEM.

**Figure 2:**
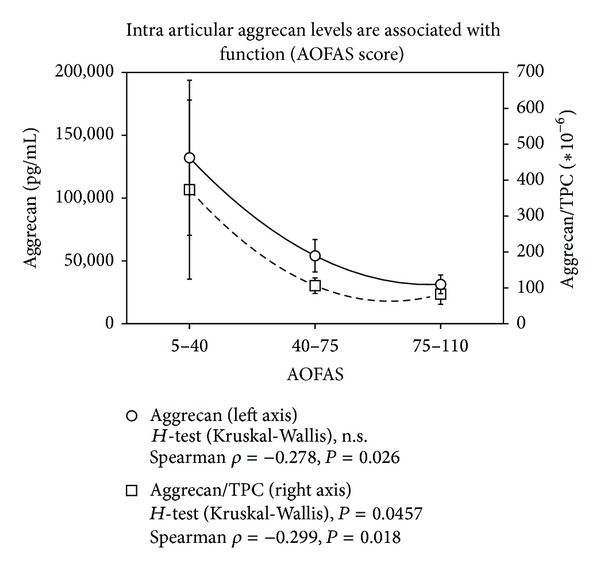
Increasing intra-articular levels of aggrecan are observed with progress of OA measured by a functional score of the ankle (AOFAS). Results of the polynomial regression analysis, the Spearman correlation, and the *post hoc* statistics (Kruskal-Wallis *H*-Test) are indicated for absolute aggrecan levels and concentrations in relation to the total protein content (TPC) on the bottom. Polynomial regression with degree of 2 gives an *R* ^2^ = 1 for absolute and for relative concentrations; values are shown with ±SEM.

**Figure 3:**
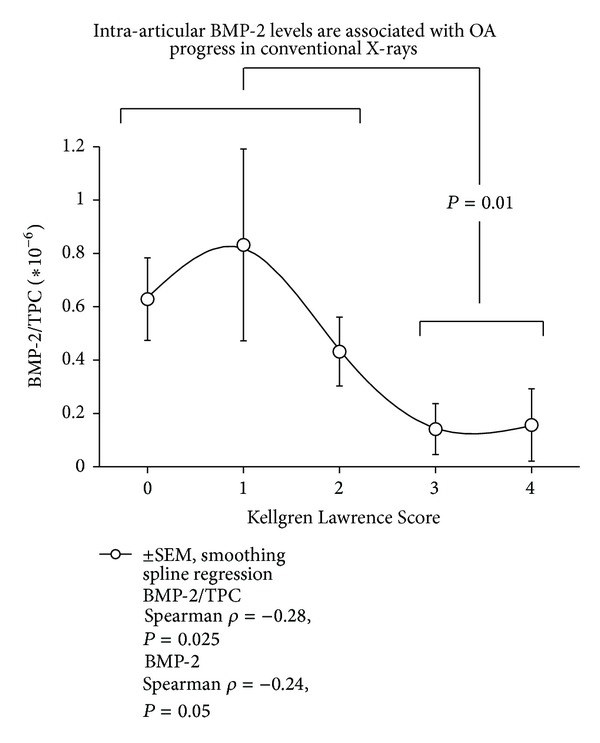
High levels of BMP-2 are associated with a low degree of OA related radiological changes (Kellgren Lawrence Score). Results of the Spearman correlation are indicated for absolute BMP-2 levels and concentrations in relation to the total protein content (TPC) on the right side. Statistical significance (Kruskal-Wallis *H*-Test) describes results comparing mild (KLS 0–2) and severe OA (KLS 3-4). A smoothing spline regression for plane curves was chosen in order to illustrate the S-shaped character of the curve; values are shown with ±SEM.

**Figure 4:**
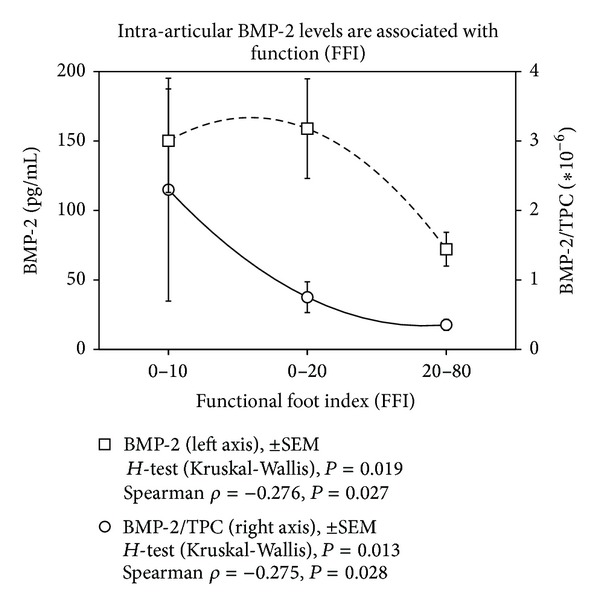
High levels of BMP-2 are associated with a good function (low FFI). Results of the Spearman correlation and the *post hoc* statistics (Kruskal-Wallis *H*-Test) are indicated for absolute BMP-2 levels and concentrations in relation to the total protein content (TPC) on the bottom. Polynomial regression with degree of 2 gives an *R* ^2^ = 1 for absolute and for relative concentrations; values are shown with ±SEM.

The mean total protein content (TPC) increased with KLS (0: 362.8 ± 296.7 *μ*g/mL, 1: 519.1 ± 484.5 *μ*g/mL, 2: 485.3 ± 302.5 *μ*g/mL, 3: 736.9 ± 823.9 *μ*g/mL, 4: 1517.9 ± 1580.7 *μ*g/mL). For correlation analysis, cytokine levels were used as absolute concentrations and concentrations in relation to TPC in order to minimize a possible dilution bias. As parameters characterizing OA duration of symptoms, functional scores and the radiological scores KLS and AOSS were used. The results of the correlation analysis are listed in Table 2. The inflammatory parameters MMP-13 and IL-1*β* did not show any statistically significant association with factors indicating progress of OA. At first, aggrecan was identified as reliable indicator of OA progress with statistically significant associations (*P* < 0.02) with duration of symptoms, FFI or AOFAS, and KLS. The data are supported by the *post hoc* statistics (Kruskal-Wallis *H*-Test) confirming a statistical association between KLS and aggrecan (Figure 1, *P* < 0.03) and AOFAS and aggrecan (3 classes, Figure 2, *P* < 0.05). Similarly, BMP-7 was related to OA progress reflected by the statistically significant correlations to duration of complains, age, FFI, AOFAS, and KLS (*P* < 0.04). This could be confirmed in case of FFI by the Kruskal-Wallis *H*-test (2 classes, FFI <47.5-good function 25.10 ± 14.81 pg/mL versus FFI >47.5-bad function 62.86 ± 55.02 pg/mL, *P* = 0.018). While aggrecan and BMP-7 levels increased with OA severity, BMP-2 levels decreased showing statistically significant negative correlations to intra-articular aggrecan or BMP-7 concentrations and ICRS score (*P* < 0.001, *ρ* = −0.48). These data are in line with the negative association of BMP-2 levels with FFI and KLS (*P* < 0.03, Table 2) and the positive correlation to endoglin levels (*P* = 0.03, *ρ* = −0.27), which is part of the BMP-receptor complex. When comparing the mean concentrations of BMP-2 in relation to TPC in the groups of KLS 0–2 (no or mild OA) and KLS 3-4 (severe OA), a statistically significant difference could be confirmed (0.67 ± 1.16 versus 0.15 ± 0.20, Figure 3, *P* = 0.01). Similarly, a very asymmetric distribution of BMP-2 levels could be observed in case of function. Only very high FFI levels (bad function) were associated with low intra-articular BMP-2 concentrations as confirmed by the Kruskal-Wallis *H*-Test (Figure 4, *P* < 0.03).

## 4. Discussion

The presented data confirm typical associations between markers of OA progress as radiographic changes or a poor clinical function with risk factors for OA as age, BMI, duration of symptoms, degree and size of cartilage damage, or stage of OCD. This has been described in a variety of publications in the background of a fracture followup [2] or OCD [36]. Based on valid clinical data, we were able to identify synovial biochemical key markers of beginning OA as aggrecan and BMP-7 in the ankle with a direct statistically significant correlation to radiographic changes and clinical outcome.

Aggrecan is a cartilage specific, large proteoglycan, and represents an integral part of the ECM. Breakdown of cartilage and matrix apparently leads to liberation of total or spliced aggrecan molecules into the articular joint space. Since progress of OA is hallmarked by a general loss of cartilage elevated intra-articular aggrecan levels are expected and truly have been described before [37]. Our data now confirm that the increase in synovial aggrecan concentration is also observed in the ankle and correlates not only with the Kellgren Lawrence Score but also with clinical function. Synovial concentrations of aggrecan have been previously shown to depend not only on progress of ankle degeneration but also on age, because concentrations were lower in joints of children and adolescents compared with adults [30]. Interestingly, the concentrations of MMP-13, an ECM degrading enzyme, did not show statistically significant associations with OA characterizing parameters. This may be explained by the fact that MMP-13 upregulation starts with an acute impact and triggers the beginning of ECM breakdown but then is downregulated again. But the process of matrix degradation is ongoing and may just be maintained by the influence of mechanical load. Whereas aggrecan seems to be a more reliable marker for chronic OA, MMP-13 may indicate the consequences of recent and acute affections of joints. We suppose a similar regulation for IL-1*β*, because we have observed similar regulation patterns and found upregulation following operations in the knee [31].

A role for BMP-7 in OA has been suspected before, because both elevated synovial and plasma levels were found to correlate with severity of disease in knee OA [38]. The anabolic effects of BMP-7 were even thought to be used in clinical trials when the recombinant protein was intra-articularly injected [39]. The introduced study now provides evidence for a similar regulation of BMP-7 in the ankle, which appears to be interesting against the background of profound differences in chondrogenic metabolism comparing cartilage from different origins [4]. The reason may probably be seen in the very different biomechanics with different degrees of load and symmetry [40, 41].

The role of BMP-2 in OA is more controversially discussed. Previously, we described reduced expression of BMPR-1A in degenerated cartilage of knees in OCD patients based on a histological analysis [25]. However, we found elevated synovial BMP-2 levels following surgical cartilage repair [31]. Operations are associated with temporarily elevated levels of IL-1*β*; and the induction of BMP-2 expression by proinflammatory conditions has been described *in vitro* too [35]. Both facts indicate regulatory differences between an acute impact inducing OA and the chronic course of the disease for regulation of BMP-2 and IL-1*β*. Therefore, interpretation of the presented results needs to keep in mind that in the presented population there was always a significant period of time between onset of symptoms and operation, during which the lavage was collected. The majority of the recruited patients of this study may be considered to be in a chronic state of mainly mild OA, explaining the missing association of OA characterizing parameters with IL-1*β* or MMP-13 and the reduced BMP-2 levels in the advanced OA stages. Similarly, in a study looking for TNF*α* levels in knees with OA a correlation to KLS was missing [42]. Since chondrocytes significantly contribute to BMP-2 secretion [21] the observed decrease in synovial levels might just be a matter of a diminished number of BMP-2 producing cells. Also, inflammation induced BMP-2 production might just be subsided as described by Dell'Accio et al. [21]. Since cartilage loss is associated with a decrease in clinical results the correlation of diminished BMP-2 levels with impaired function becomes plausible. The high asymmetry in the found BMP-2 regulation further supports this hypothesis, because synovial BMP-2 levels slightly increased with KLS to asymptotically fall down in an S-shaped matter at KLS stages 3 and 4.

Limitations of the study are the low case number and the fact that OCD predominates the selected OA causes. Although only 49 patients could be included all found associations were confirmed in different associations, which indicates a reliable soundness of the data. Since this study had pilot character and associations or concentrations were not published before, an initial power calculation was not possible. Therefore, the results do not have confirmatory character. Although OA may be caused by different pathological triggers in the beginning of disease there seems to be a common final path. Therefore, it may be suspected that at a certain point it does not matter which reason initially started the osteoarthritic development in the ankle, because the resulting biochemistry will be equalized caused by the same alterations as loss of cartilage, formation of osteophytes, and sclerosis of the subchondral bone.

Summarizing, on the basis of valid clinical data we were able to identify key markers of chronic OA in the ankle as aggrecan and BMP-7, both increasing with OA progress. In contrast, high levels of BMP-2 were associated with a good clinical function and low signs of OA related radiographic changes. Together with better understanding of OA biochemistry in the ankle this knowledge offers starting points for possible new ways in diagnostics and interventional strategies.

## Figures and Tables

**Table 1:** Correlation of items characterizing a cartilage lesion in the ankle with epidemiological data, function, and radiological parameters.

	Age	Gender*	BMI	FFI	CFSS	AOFAS	KLS	AOSS
ICRS score								
Corr. coefficient	0.2654	0.0995	0.2179	0.4382	−0.3780	−0.3105	0.4777	0.6557
Valid cases	49	49	49	49	49	49	49	38
Significance (*P*)	0.0327	n.s.	n.s.	0.0008	0.0037	0.0149	0.0002	3.939*E* − 06
Defect size								
Corr. coefficient	0.1199	0.0038	0.0928	0.2955	−0.3726	−0.2309	0.3733	0.7145
Valid cases	31	31	31	31	31	31	31	25
Significance (*P*)	n.s.	n.s.	n.s.	n.s.	0.0195	n.s.	0.0193	3.002*E* − 05
Previous operation*								
Corr. coefficient	0.2943	0.2306	0.1460	0.3493	−0.3107	−0.2413	0.3594	0.5547
Valid cases	49	49	49	49	49	49	49	38
Significance (*P*)	0.0200	n.s.	n.s.	0.0069	0.0149	0.0475	0.006	0.0001
Duration of complains								
Corr. coefficient	0.3925	0.25377	0.1813	0.2638	−0.2336	−0.2849	0.4368	0.3917
Valid cases	49	49	49	49	49	49	49	38
Significance (*P*)	0.0026	0.0392	n.s.	0.0335	n.s.	0.0236	0.0008	0.0075

BMI: body mass index, FFI: foot function index, CFSS: calcaneal fractures scoring system according to Kerr, AOFAS: ankle-hindfoot scale, KLS: Kellgren Lawrence Score, AOSS: ankle osteoarthritis scoring system, corr. coefficient: correlation coefficient Spearman's *P*, *U-test.

**Table 2:** Correlation of intra-articular protein levels with duration of complains, functional scores, and OA-related changes in conventional X-ray or MRI.

	Duration of complains	FFI (reverse)	CFSS	AOFAS	KLS	AOSS
MMP-13/MMP-13/TPC						
Correlation	—/—	—/—	—/—	—/—	—/—	—/—
Valid cases	49	49	49	49	49	38
Signif. (*P*)	n.s./n.s.	n.s./n.s.	n.s./n.s.	n.s./n.s.	n.s./n.s.	n.s./n.s.
IL-1*β*/IL-1*β*/TPC						
Correlation	—/—	—/—	—/—	—/—	—/neg.	—/—
Valid cases	49	49	49	49	49	38
Signif. (*P*)	n.s./n.s.	n.s./n.s.	n.s./n.s.	n.s./n.s.	n.s./0.023	n.s./n.s.
bFGF/bFGF/TPC						
Correlation	pos./—	—/—	—/—	—/—	pos./—	—/—
Valid cases	49	49	49	49	49	38
Signif. (*P*)	0.043/n.s.	n.s./n.s.	n.s./n.s.	n.s./n.s.	0.044/n.s.	n.s./n.s.
BMP-2/BMP-2/TPC						
Correlation	—/—	pos./pos.	—/—	—/—	—/neg.	—/—
Valid cases	49	49	49	49	49	38
Signif. (*P*)	n.s./n.s.	0.027/0.028	n.s./c	n.s./n.s.	n.s./0.025	n.s./n.s.
BMP-7/BMP-7/TPC						
Correlation	pos./—	pos./—	—/—	neg./—	pos./—	—/—
Valid cases	49	49	49	49	49	38
Signif. (*P*)	0.014/n.s.	0.008/n.s.	n.s./n.s.	0.032/n.s.	0.014/—	n.s./n.s.
Endoglin/endoglin/TPC						
Correlation	—/—	—/—	pos./—	—/—	—/—	—/—
Valid cases	49	49	49	49	49	38
Signif. (*P*)	n.s./n.s.	n.s./n.s.	0.025/n.s.	n.s./n.s.	n.s./n.s.	n.s./n.s.
IGF-1/IGF-1/TPC						
Correlation	—/—	neg./—	pos./—	—/—	—/—	—/—
Valid cases	49	49	49	49	49	38
Signif. (*P*)	n.s./n.s.	0.042/n.s.	0.031/n.s.	n.s./n.s.	n.s./n.s.	n.s./n.s.
IGF-1R/IGF-1R/TPC						
Correlation	—/—	—/neg.	—/—	—/—	—/neg.	—/—
Valid cases	49	49	49	49	49	38
Signif. (*P*)	n.s./n.s.	n.s./0.037	n.s./n.s.	n.s./n.s.	n.s./0.010	n.s./n.s.
Aggrecan/aggrecan/TPC						
Correlation	pos./pos.	pos./pos.	—/—	neg./neg.	pos./pos.	—/—
Valid cases	49	49	49	49	49	38
Signif. (*P*)	0.004/0.010	0.036/0.022	n.s./n.s.	0.026/0.018	0.001/0.005	n.s./n.s.

Signif. (*P*): *P* value for statistical significance Spearman's *P*, FFI: foot function index, CFSS: calcaneal fractures scoring system according to Kerr, AOFAS: ankle-hindfoot scale, KLS: Kellgren Lawrence Score, AOSS: ankle osteoarthritis scoring system. Statistical tests refer to correlations to the absolute protein concentrations and the protein concentrations in relation to TPC.
